# Construction of survival prediction model for elderly esophageal cancer

**DOI:** 10.3389/fonc.2022.1008326

**Published:** 2022-10-19

**Authors:** Shuai Qie, Hongyun Shi, Fang Wang, Fangyu Liu, Jinling Gu, Xiaohui Liu, Yanhong Li, Xiaoyue Sun

**Affiliations:** ^1^ Department of Radiation Oncology, Affiliated Hospital of Hebei University, Baoding, China; ^2^ Department of Radiation Oncology, Baoding First Central Hospital, Baoding, China

**Keywords:** esophageal cancer, SEER, nomogram, survival analysis, elderly

## Abstract

**Background:**

The purpose of this study was to analyze the clinical characteristics and prognosis of EPEC and to construct a prediction model based on the SEER database.

**Methods:**

All EPECs from the SEER database were retrospectively analyzed. A comprehensive and practical nomogram that predicts the overall survival (OS) of EPEC was constructed. Univariate and multivariate Cox regression analysis was performed to explore the clinical factors influencing the prognosis of EPEC, and finally, the 1 -, 3 - and 5-year OS were predicted by establishing the nomogram. The discriminant and predictive ability of the nomogram was evaluated by consistency index (C-index), calibration plot, area under the curve (AUC), and receiver operating characteristic (ROC) curve. Decision curve analysis (DCA) was used to evaluate the clinical value of the nomogram.

**Results:**

A total of 3478 patients diagnosed with EPEC were extracted from the SEER database, and the data were randomly divided into the training group (n=2436) and the validation group (n=1402). T stage, N stage, M stage, surgery, chemotherapy, radiotherapy, age, grade, and tumor size were independent risk factors for 1 -, 3 - and 5-year OS of EPEC (P< 0.05), and these factors were used to construct the nomogram prediction mode. The C-index of the validation and training cohorts was 0.718 and 0.739, respectively, which were higher than those of the TNM stage system. The AUC values of the nomogram used to predict 1-, 2-, and 3-year OS were 0.751, 0.744, and 0.786 in the validation cohorts (0.761, 0.777, 0.787 in the training cohorts), respectively. The calibration curve of 1-, 2-, and 3-year OS showed that the prediction of the nomogram was in good agreement with the actual observation. The nomogram exhibited higher clinical utility after evaluation with the 1-, 2-, and 3-year DCA compared with the AJCC stage system.

**Conclusions:**

This study shows that the nomogram prediction model for EPEC based on the SEER database has high accuracy and its prediction performance is significantly better than the TNM staging system, which can accurately and individually predict the OS of patients and help clinicians to formulate more accurate and personalized treatment plans.

## Introduction

Esophageal cancer has become one of the most common malignant tumors in the world. According to global cancer statistics in 2020, the number of new cases of esophageal cancer reached 604,000 and the number of deaths reached 544,000 (1). The incidence of esophageal cancer in the aged gradually increases with the aging of the population (2). Most elderly patients often difficult to accept surgical treatment due to a lot of past medical history, organ function decline, poor physical condition, and other reasons, and even give up chemotherapy and choose radiotherapy as its radical treatment (3). Diabetes and hypertension are common medical diseases in the elderly, and their incidence continues to increase. There are few clinical studies on whether these basic diseases have an impact on the toxic side effects and efficacy of radiotherapy (4). Symptoms appear at an advanced stage due to a general lack of responsiveness in the elderly.

The prognostic factors of EPEC are still controversial. Currently, the TNM (Tumor-Node-Metastasis) staging system is considered the most widely used prognostic assessment system and clinical treatment of cancer patients, but it only includes the depth of local tumor invasion, the range of regional lymph node metastasis, and the state of distant metastasis (5). However, many important clinical features may potentially affect the prognosis of esophageal cancer. Therefore, the main aim of this study is to develop richer and more accurate prognostic models to guide survival.

The alignment diagram, also known as the nomogram diagram, is based on multi-factor regression analysis, integrating multiple prediction factors and drawing them in a certain proportion on the same plane with graduated line segments, so as to express the relationship between variables in the prediction model. In this study, based on the data of the SEER (Surveillance, Epidemiology, and End Results) database, the clinicopathological features affecting the prognosis of EPEC were discussed for the first time and the prognostic variables were further studied. Finally, we further construct a nomogram model to predict the prognosis of EPEC.

## Methods

### Patients selection and data acquisition

The study was based on clinical data from 18 (SEER) cancer registries. In this study, SEER*Stat software (version 8.4) was used to search the SEER database for patients older than 65 years of age with primary esophageal cancer from 2010 to 2015. Inclusion criteria of this study: (I) Primary esophageal cancer; (II) The years of diagnosis were from 2010 to 2015; (III) Single primary tumor; (IV) Pathological diagnosis is clear; (V). Older than 65. Exclusion criteria: (I) No follow-up time; (II) Incomplete data; (III) Younger than 65 years old. All data in this study were extracted from the SEER database free of charge.

### Statistical analysis

Statistical analysis was performed using SPSS 25.0 software and R language 3.6.1. Patients were randomly divided into training set and validation set by 7∶3 to construct this nomogram. The cut-off values of continuous variables were determined by X-tile software and converted into classified variables. We performed a descriptive analysis of the clinical baseline data of the enrolled patients and used the Chi-square test to compare the characteristics of patients in the training and validation groups. COX hazard ratio model was used to analyze the factors influencing the survival and prognosis of patients in the training set. Factors of P<0.05 were included in the multifactor analysis to determine the final independent prognostic factors, and the nomogram containing these independent prognostic factors was constructed using R language. Internal and external validation was carried out in the training set and validation set, respectively.

The prediction effect of this model is evaluated by the area under (AUC) the receiver operating characteristic curve (ROC). The discriminative power of the model was evaluated by the concordance index (C-index). The clinical utility was analyzed using a decision curve analysis (DCA). DCA represents the net benefit of clinical decision-making. The Y-axis represents the net benefit and the X-axis represents the risk threshold. P < 0.05 was considered statistically significant.

## Results

### Baseline characteristics

**Figure 1:**
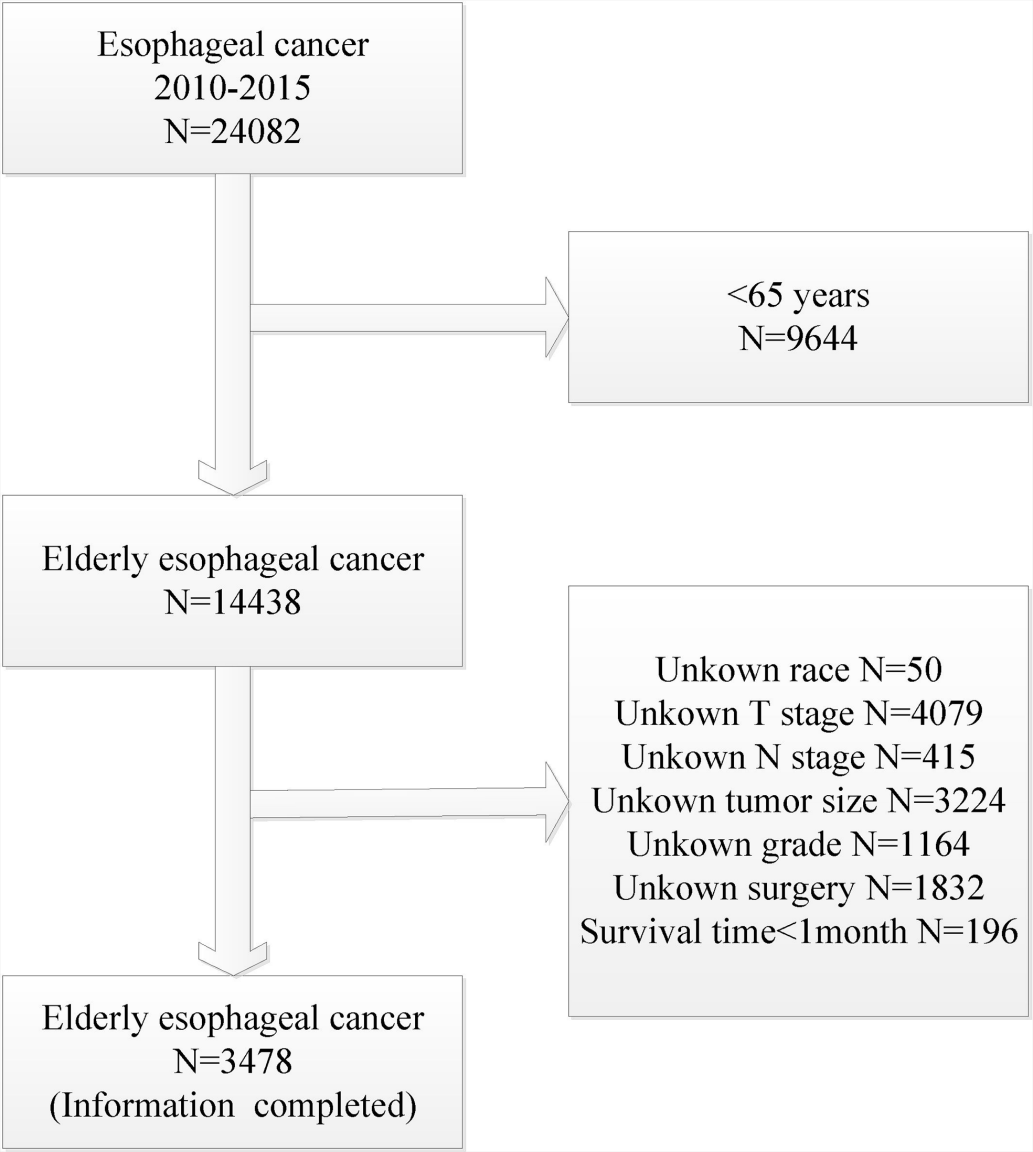
Flow diagram of selecting process.

Sex, age, race, T stage, N stage, M stage, pathological type, radiotherapy, chemotherapy, surgery, tumor location, pathological grade, and tumor size were included in the analysis. According to the inclusion and exclusion criteria, a total of 3478 eligible patients were screened from the SEER database between 2010 and 2015. A complete flow chart describing the selection process is shown on **Figure 1**. One-third of the patients were randomly assigned to the validation group and the rest were used to construct the nomogram prediction model. The detailed clinicopathological features of all cases were shown in **Table 1**.

**Table 1:** Demographics and characteristics of patients in Training and Validation cohorts.

		Training group (n=2436)	Validation group (n=1042)	χ2	P
Age				0.846	0.665
	>83	239	112		
		9.80%	10.70%		
	65-71	1115	478		
		45.80%	45.90%		
	72-83	1082	452		
		0.444	0.434		
Sex				1.223	0.269
	Female	539	213		
		22.10%	20.40%		
	Male	1897	829		
		77.90%	79.60%		
Race				1.985	0.371
	Black	161	71		
		6.60%	6.80%		
	Other	126	66		
		5.20%	6.30%		
	White	2149	905		
		88.20%	86.90%		
T stage				2.715	0.438
	T1	730	305		
		30.00%	29.30%		
	T2	323	138		
		13.30%	13.20%		
	T3	1071	483		
		44.00%	46.40%		
	T4	312	116		
		12.80%	11.10%		
N stage				14.028	0.003
	N0	1069	388		
		43.90%	37.20%		
	N1	996	475		
		40.90%	45.60%		
	N2	280	129		
		11.50%	12.40%		
	N3	91	50		
		3.70%	4.80%		
M stage				2.031	0.154
	M0	1966	819		
		80.70%	78.60%		
	M1	470	223		
		19.30%	21.40%		
Histology				4.312	0.116
	ADC	1562	659		
		64.10%	63.20%		
	Other	107	63		
		4.40%	6.00%		
	SCC	767	320		
		31.50%	30.70%		
Grade				0.317	0.957
	I	161	68		
		6.60%	6.50%		
	II	1030	451		
		42.30%	43.30%		
	III	1203	506		
		49.40%	48.60%		
	IV	42	17		
		1.70%	1.60%		
Primary site				1.658	0.798
	Abdominal	1590	691		
		65.30%	66.30%		
	Cervical	154	60		
		6.30%	5.80%		
	NOS	100	38		
		4.10%	3.60%		
	Overlapping	106	39		
		4.40%	3.70%		
	Thoracic	486	214		
		20.00%	20.50%		
Tumor size				0.106	0.948
	<39	916	386		
		37.60%	37.00%		
	>62	608	261		
		25.00%	25.00%		
	39-62	912	395		
		37.40%	37.90%		
Surgery				3.896	0.048
	No	1561	704		
		64.10%	67.60%		
	Yes	875	338		
		35.90%	32.40%		
Radiotherapy				0.290	0.590
	No	814	358		
		33.40%	34.40%		
	Yes	1622	684		
		66.60%	65.60%		
Chemotherapy				0.197	0.657
	No	769	321		
		31.60%	30.80%		
	Yes	1667	721		
		68.40%	69.20%		

ADC, adenocarcinoma; SCC, Squamous cell carcinomas.

### Independent predictors in the study population

The cut-off values of continuous variables were determined by X-tile software and converted into classified variables. Univariate Cox regression analysis showed that the factors influencing the prognosis of old esophageal cancer patients were race, tumor site, T stage, N stage, M stage, surgery, chemotherapy, radiotherapy, age (65-71 years, 72-83 years, and >83 years), histology, grade, and tumor size (<39mm, 39-62mm, and >62mm). The above 12 factors were again included in the multivariate Cox regression analysis, and the results showed that T stage, N stage, M stage, surgery, chemotherapy, radiotherapy, age, grade, and tumor size were independent factors influencing the prognosis of old esophageal cancer (**Table 2**).

**Table 2:** Univariate and multivariate Cox analysis of prognostic factors.

Variable	Univariate analysis					Multivariate analysis			
	Count	HR	95% CI		p-value	HR	95% CI		p-value
**Sex**					0.659				
female	539	Reference							
male	1897	1.026	0.915	1.152					
Race			0.928		0.005				0.276
black	161	Reference				Reference			
other	126	0.892	0.684	1.164	0.401	1.083	0.828	1.418	
white	2149	0.759	0.633	0.909	0.003	0.921	0.758	1.12	
**Site**					< 0.001				0.501
Abdominal	1590	Reference				Reference			
Cervical	154	1.349	1.119	1.627	0.002	0.952	0.772	1.173	
NOS	100	1.504	1.2	1.885	< 0.001	1.188	0.942	1.497	
Overlap	106	1.43	1.144	1.788	0.002	1.007	0.797	1.271	
Thoracic	486	1.124	0.997	1.268	0.057	0.948	0.825	1.088	
**T stage**					< 0.001				< 0.001
T1	730	Reference				Reference			
T2	323	0.859	0.727	1.014	0.073	0.993	0.835	1.181	0.929
T3	1071	1.186	1.057	1.331	0.004	1.165	1.021	1.331	0.026
T4	312	2.391	2.058	2.778	< 0.001	1.49	1.265	1.756	< 0.001
**N stage**					< 0.001				
N0	1069	Reference				Reference			< 0.001
N1	996	1.484	1.336	1.648	< 0.001	1.249	1.112	1.403	< 0.001
N2	280	1.749	1.503	2.035	< 0.001	1.643	1.394	1.935	< 0.001
N3	91	2.317	1.832	2.93	< 0.001	1.636	1.276	2.097	< 0.001
**M stage**					< 0.001				< 0.001
M0	1966	Reference				Reference			
M1	470	2.864	2.562	3.201		1.731	1.523	1.968	
**Surgery**					< 0.001				< 0.001
No	1561	Reference				Reference			
Yes	875	0.3	0.268	0.336		0.37	0.323	0.422	
**Chemotherapy**					< 0.001				< 0.001
No	769	Reference				Reference			
Yes	1667	0.727	0.658	0.805		0.487	0.43	0.552	
**Radiotherapy**					0.005				0.029
No	814	Reference				Reference			
Yes	1622	0.865	0.783	0.957		0.869	0.767	0.986	
**Age**					< 0.001				< 0.001
>83	239	Reference				Reference			
65-71	1115	0.454	0.389	0.53	< 0.001	0.675	0.571	0.797	0.083
72-83	1082	0.659	0.566	0.767	< 0.001	0.868	0.741	1.017	< 0.001
**Histology**					< 0.001				0.375
ADC	1562	Reference				Reference			
Other	107	1.211	0.964	1.523	0.1	1.13	0.899	1.419	
SCC	767	1.247	1.127	1.38	< 0.001	1.076	0.945	1.225	
**Grade**					< 0.001				0.001
I	161	Reference				Reference			
II	1030	1.297	1.043	1.613	0.019	1.109	0.89	1.382	0.37
III	1203	1.722	1.389	2.134	< 0.001	1.324	1.063	1.649	0.015
IV	42	2.221	1.504	3.28	< 0.001	1.515	1.014	2.263	0.049
**T size**					< 0.001				< 0.001
< 39	916	Reference				Reference			
>62	608	2.382	2.106	2.695	< 0.001	1.632	1.424	1.871	< 0.001
39-62	912	1.769	1.578	1.983	< 0.001	1.364	1.206	1.544	< 0.001

ADC, adenocarcinoma; SCC, Squamous cell carcinomas.

### Prognostic nomogram building and validation

**Figure 2:**
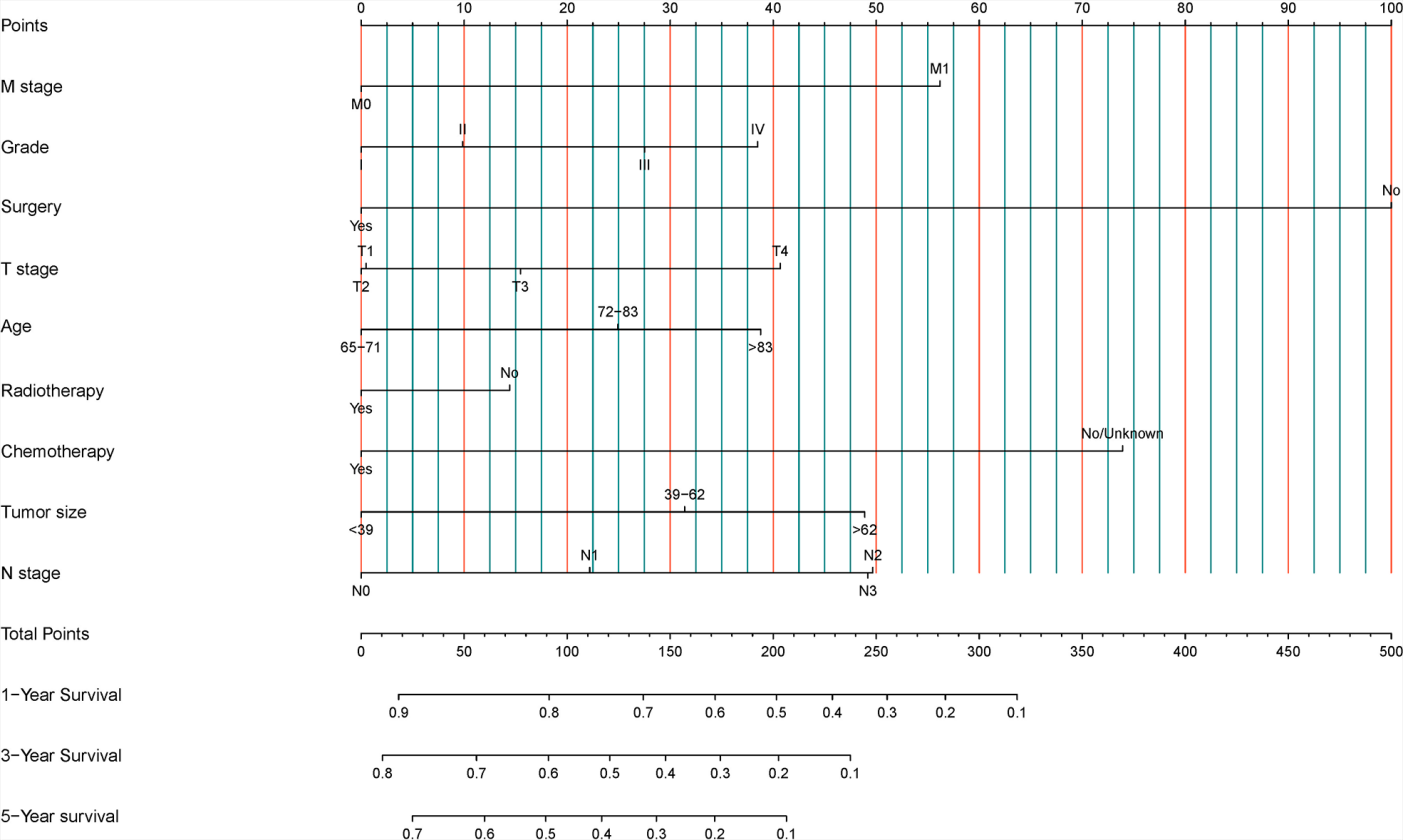
Development of a prognostic stratification nomogram and validation of the proposed nomogram.

Nine statistically significant independent prognostic factors were included in the above multivariate COX proportional regression model to construct a nomogram to predict 3-year and 5-year overall survival (**Figure 2**). Individual scores can be read for each clinicopathological indicator in each patient, and the scores are added together to obtain an overall score.

Compared with the AJCC staging system, the C-index of the training cohorts and validation cohorts were 0.739 (95%CI: 0.727~0.750) and 0.718 (95%CI: 0.700~0.736), respectively, indicating that the nomogram had the good predictive ability, while C-indices of the AJCC stage system were 0.642 (95% CI: 0.627–0.656) and 0.630 (95% CI: 0.608–0.653) in the training cohorts and the validation cohorts, respectively.

**Figure 3:**
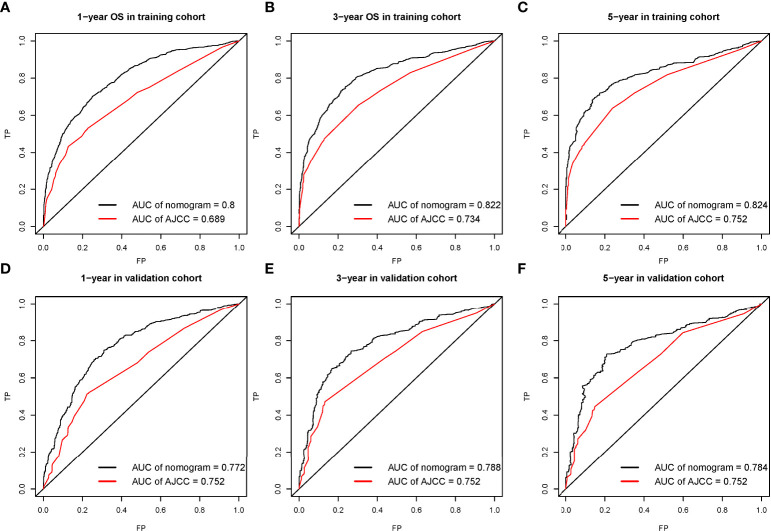
The receiver operating characteristic (ROC) curve for nomogram in the training cohort **(A–C)** and validation cohort **(D–F)** at 1-year, 3-year, and 5-year, respectively.

For OS, this study draws the area under the ROC curve (AUC) of the nomogram prediction model and TNM staging system (as shown in **Figure 3**), which intuitively shows the performance of the nomogram prediction model is better than that of the TNM staging system. In the training cohorts, the 1-, 3-, and 5-year OS of AUC of this nomogram was superior to that of the AJCC stage system (1-year OS AUC: 0.8 vs. 0.689, 3-year OS AUC: 0.822 vs. 0.734, 5-year OS AUC: 0.824 vs. 0.752, respectively, **Figures 3A–C**), whereas the AUC of this nomogram and the AJCC stage system is shown in **Figures 3D–F** for the validation cohorts (1-year OS AUC: 0.772 vs. 0.752, 2-year OS AUC: 0.788 vs. 0.752, 3-year OS AUC: 0.784 vs. 0.752).

**Figure 4:**
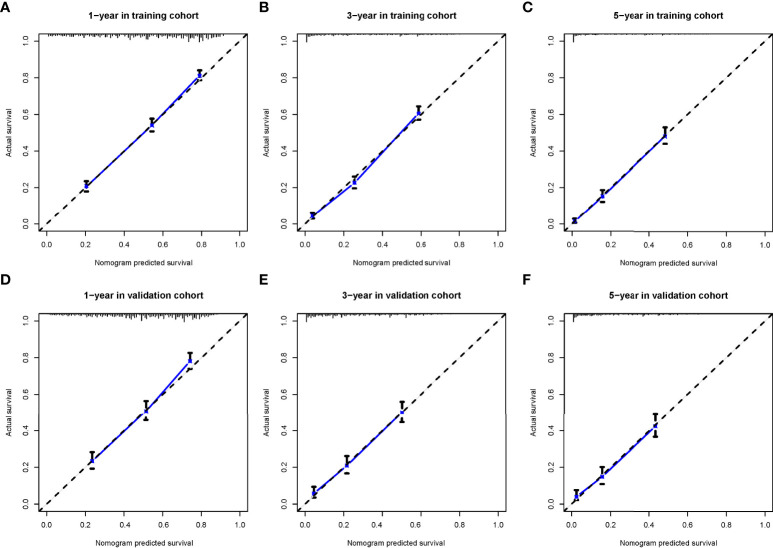
The calibration curves for predicting patients’ overall survival in the training cohort **(A–C)** and validation cohort **(D–F)** at 1-year, 3-year, and 5-year, respectively.

The calibration curve shows that there was a high degree of agreement between the nomogram prediction and the actual 1-, 3-, and 5-year OS in the training cohorts (**Figures 3A–C**) and the validation cohorts (**Figures 4D–F**).

### Differences in the nomogram and the 7th AJCC TNM stage system

**Figure 5:**
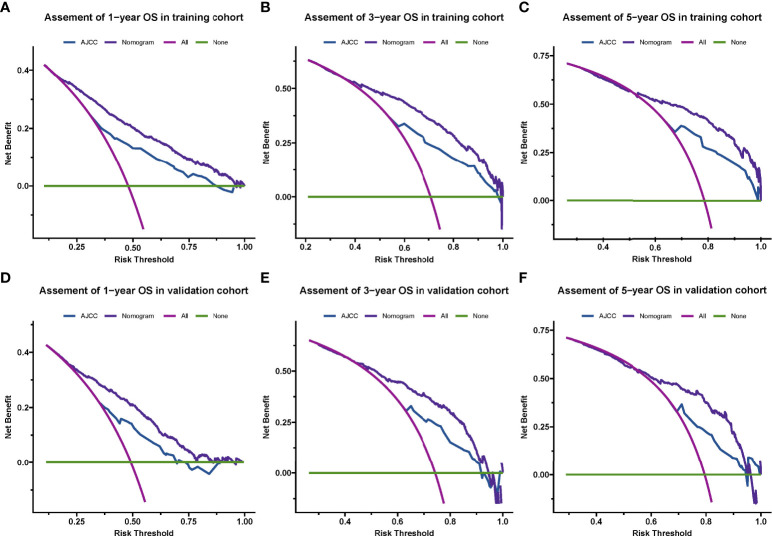
Decision curve analysis for the nomogram and AJCC stage in the training cohort **(A–C)** and validation cohort **(D–F)** at 1-year, 3-year, and 5-year, respectively.

By drawing a decision Curve analysis (DCA) diagram (as shown in **Figure 4**) to further compare the clinical application value of the Nomogram prediction model with the TNM staging system, it is found that in almost all threshold probabilities at different points, The net return of Nomogram prediction model is better than TNM staging system, showing better clinical efficacy of the new model (**Figure 5**).

### Risk stratification model and survival analysis

**Figure 6:**
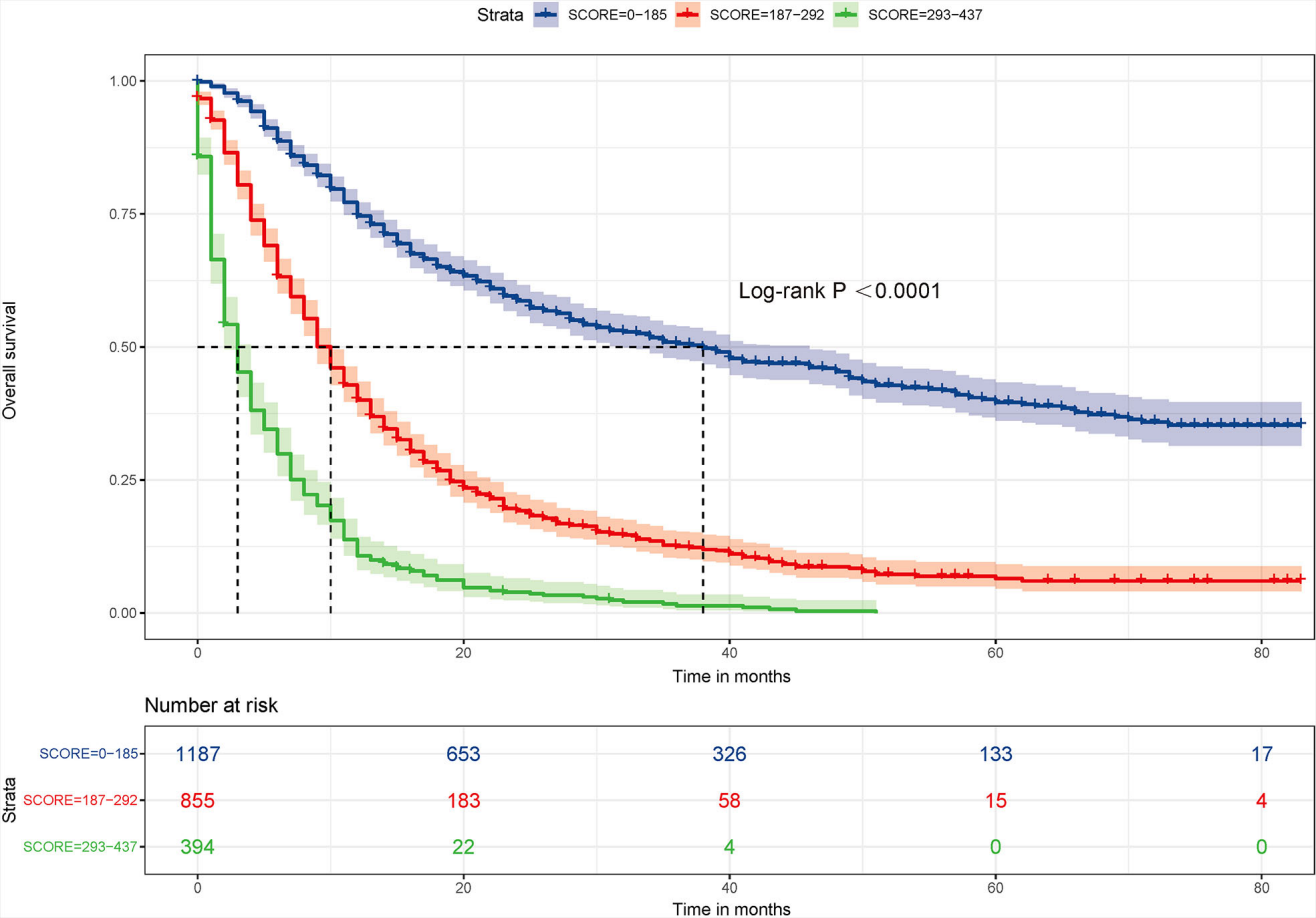
Survival benefit of surgery in the low-risk, intermediate-risk, and high-risk groups.

For each variable in this nomogram, a total score is calculated for each patient and divided into 3 levels: low-risk (scores 0-185), intermediate-risk (scores 186-292), and high-risk (scores 293-437) group. Kaplan-Meier curves (**Figure 6**) show that this nomogram prediction ability is excellent and risk stratification is accurate.

## Discussion

The incidence of esophageal cancer began to rise rapidly after the age of 45, and with the increase of age, the incidence of esophageal cancer increased and reached a peak between 80 and 84 years old (6). Multiple retrospective analyses found that postoperative complications in elderly patients with esophageal cancer increased significantly, tolerance decreased, and perioperative mortality increased (7). For operable esophageal cancer, patients over 70 years old should be comprehensively evaluated before surgery (8). Patients with high surgical risk, complications, and poor cardiopulmonary function can be treated with radical radiotherapy. Radical radiotherapy is the main treatment for inoperable senile esophageal carcinoma (9). There are few reports on the results of high-grade randomized studies on EPEC only. Randomized clinical trials typically exclude patients over 70 years of age from esophageal cancer (10). Therefore, the present about the elderly esophagus. Most of the data on radiotherapy and chemotherapy for cancer come from retrospective studies, the number of cases is generally small, and the treatment standard has not been unified.

The TNM staging system is the most commonly used tumor staging system in the world, which helps doctors understand the progress of cancer, and can help doctors make treatment plans and judge the prognosis (11). Oncologists and patients alike want reliable prognostic information for each patient. The nomogram is more advantageous than the traditional TNM staging system, so it has been proposed as an alternative or even a new standard (12). The personalized predictive power of the nomogram allows it to be used to identify and stratify patients participating in clinical trials. The combination of friendly interfaces and extensive web availability makes them popular among oncologists and patients (13).

In this retrospective study, independent prognostic factors affecting survival in EPEC were obtained through univariate and multivariate analyses of SEER database data. Compared with the AJCC staging system, we constructed a new visual nomogram using these independent prognostic factors to predict the 1-, 3-, and 5-year overall survival with higher accuracy. The results of this study showed that T stage, N stage, M stage, tumor grade, tumor size, patient age, surgical status, radiotherapy status, and chemotherapy status were independent prognostic factors affecting EPEC.

With the improvement of esophageal surgery theory and technology, anesthesia technology, perioperative management, and the development and improvement of related disciplines and equipment, the surgical treatment effect of esophageal cancer has made great progress, and the safety factor of surgery has been greatly improved (14). Therefore, most scholars believe that surgery can completely remove the tumor, and as long as the patient can tolerate it, surgical treatment should be the first choice, and age should not be a limit for surgical treatment of esophageal cancer (15–17). Because elderly patients are often complicated with cardiovascular, cerebrovascular, and respiratory diseases, it is often believed that there are more postoperative complications, including surgery-related and non-surgery-related complications, which increase the perioperative mortality. The results of Tanja M (18) showed that there was no significant difference between the elderly patients (≥70 years old) and the young patients (< 70 years old) with surgery-related complications, which were 20% and 17%, respectively. The results of this study show that surgery can significantly prolong the overall survival of EPEC, which is consistent with the published literature.

To date, there are no guidelines for the treatment of EPEC. RTOG8501 compared the efficacy of 50Gy combined with cisplatin and fluorouracil combined with concurrent chemoradiotherapy plus chemotherapy and 64Gv alone in patients with esophageal cancer (23% of patients aged ≥70 years), and the results showed that the efficacy of concurrent chemoradiotherapy was significantly better than that of radiotherapy alone (5-year overall survival: 26%: 0), but concurrent chemoradiotherapy also resulted in severe acute side effects (grade 3-4 hematological side effects, 48% vs. 3%; grade 3-4 upper gastrointestinal reaction 33%: 18%); Among the patients who were subsequently enrolled in the concurrent chemoradiotherapy group, the completion rate of concurrent chemotherapy was only 68%. Therefore, the effect of concurrent chemoradiotherapy is better than radiotherapy alone.

With the progress and development of radiotherapy technology, the delineation of esophageal cancer radiotherapy target should be based on simulated positioning CT and enhanced contrast agent, so as to better confirm the target location. Intensity Modulated Radiation Therapy (IMRT), which is considered to be better than three-dimensional conformal radiotherapy (19, 20), is currently widely recommended. IMRT technology has better target conformal and can reduce the dose of important organs such as the heart, lung, and other tissues. In the treatment of esophageal cancer, the long-term damage of normal tissues is an important factor affecting the survival time and quality of life of patients in the later stage. Therefore, the application of IMRT technology provides a powerful technical condition to more strictly limit the dose of lung, heart and other important organs. Throughout the studies on esophageal cancer in recent years, a number of retrospective studies suggest that IMRT technology can improve the local control rate and survival of patients compared with 3DCT technology. With the help of IMRT, the 5-year overall survival for locally advanced esophageal cancer increased from 15% to 44% (21).

In the validation group, the calibration curve shows a high degree of agreement between nomogram predicted survival and actual survival. In addition, we find that the nomogram prediction model is superior to TNM staging system in terms of consistency index (C-index), area under ROC curve (AUC), and decision curve (DCA). Furthermore, in this study we attempt by nomogram prediction model to predict the total score, according to the scores of the risk is divided into three groups, low, medium and high risk through analysis showed the accuracy of the prediction model of risk stratification, such a high layer can effectively identify, between the survival outcomes for patients with low risk, which provide decision basis for the treatment of patients with different solutions.

Nonetheless, the study has several limitations. First, there may be selection bias because we excluded patients with incomplete information about variables. Secondly, the SEER database lacks some important parameters and specific information related to prognosis, such as the family history of esophageal cancer, radiotherapy, and chemotherapy. However, the nomogram of this study has been verified internally and has excellent clinical practicability. In conclusion, the nomogram prediction model constructed in this study can accurately predict the prognosis of EPEC and is superior to the TNM clinical staging system. It is expected that this model can be helpful to pathologists and oncologists in designing clinical strategies.

## Data availability statement

The original contributions presented in the study are included in the article/Supplementary Material. Further inquiries can be directed to the corresponding author.

## Author contributions

Study concept and design, SQ and HS. Analysis and interpretation of data, FW, FL, and JG. Drafting of the manuscript, SQ and XL. Critical revision of the manuscript for important intellectual content, YL and XS. All authors contributed to manuscript revision, read, and approved the submitted version.

## Conflict of interest

The authors declare that the research was conducted in the absence of any commercial or financial relationships that could be construed as a potential conflict of interest.

## Publisher’s note

All claims expressed in this article are solely those of the authors and do not necessarily represent those of their affiliated organizations, or those of the publisher, the editors and the reviewers. Any product that may be evaluated in this article, or claim that may be made by its manufacturer, is not guaranteed or endorsed by the publisher.
